# Characterizing the Landscape of Safety Net Programs and Policies in California during the COVID-19 Pandemic

**DOI:** 10.3390/ijerph19052747

**Published:** 2022-02-26

**Authors:** Kaitlyn E. Jackson, Joseph Yeb, Wendi Gosliner, Lia C. H. Fernald, Rita Hamad

**Affiliations:** 1 Philip R. Lee Institute for Health Policy Studies, University of California San Francisco, San Francisco, CA 94158, USA; joseph.yeb@ucsf.edu (J.Y.); rita.hamad@ucsf.edu (R.H.); 2 Division of Agriculture and Natural Resources, Nutrition Policy Institute, University of California, Oakland, CA 94607, USA; wgosliner@ucanr.edu; 3 Division of Community Health Sciences, School of Public Health, University of California Berkeley, Berkeley, CA 94720, USA; fernald@berkeley.edu; 4 Department of Family & Community Medicine, University of California San Francisco, San Francisco, CA 94110, USA

**Keywords:** COVID-19, public health policy, socioeconomic factors, government, safety net, health disparities, California, economic transition

## Abstract

The COVID-19 pandemic prompted rapid and innovative policymaking around the world at the national, regional, and local levels. There has been limited work to systematically document and characterize new and expanded local U.S. pandemic-era policies, which is imperative to better understand the policy variation and resulting health impacts during this unprecedented time. California, the most populous U.S. state, provides a case example of a particularly active policy response. The aim of this Brief Report is to summarize the creation and potential areas of application of a newly created publicly available California- and US-based COVID-19 policy database. We generated an extensive list of California and US policies that were modified or created in response to the COVID-19 pandemic. From July–November 2021, we searched current and historical California and federal government websites, press releases, social media, and news sources and recorded detailed information on these policies, including coverage dates, eligibility criteria, and benefit amounts. This comprehensive dataset includes 39 public health, economic, housing, and safety net programs and policies implemented at both federal and state levels and provides details of the complex and multifaceted policy landscape in California from March 2020 to November 2021. Our database is publicly available. Future investigators can leverage the information systematically recorded in this database to rigorously assess the short- and long-term effects of these policies, which will in turn inform future preparedness response plans in California and beyond.

## 1. Introduction

The COVID-19 pandemic and associated mitigating measures have fundamentally changed daily life on a global scale. In the United States, the pandemic has further exacerbated pre-existing racial and socioeconomic disparities in health, with minoritized racial/ethnic and low-income populations more likely to be infected, die, or suffer economic consequences [1,2]. Social and economic policies have the potential to alleviate health inequities [3,4,5,6], and these most recent disparities underscore the need for policies to address disproportionate adverse effects on vulnerable groups.

Accordingly, the pandemic has prompted rapid policymaking in many countries across the globe at the national, regional, and local governmental levels [7], including the United States [8]. The state of California— the second-most diverse U.S. state [9], in which 1 in 8 Americans live—has been particularly active in its COVID-19 policy response: it was the first to implement a statewide shelter-in-place order in March 2020 and to mandate vaccines for teachers and healthcare workers in August 2021 [10]. The state has also been at the forefront of social and economic policies to prevent and address pandemic-related disparities, including the creation and expansion of numerous safety net programs [11]. This is particularly important given California’s high poverty rate (16.4%, compared with the national average of 10.5%) and the high COVID-19 death and case rates among low-income and communities of color [1,12].

These disparities highlight the urgent need for rigorous assessment of current pandemic-related programs and policies to evaluate which were most salient for vulnerable groups. Systematically documenting new and expanded pandemic-era policies can help assess policies’ health impacts. However, to our knowledge, only two publicly available databases capture variation in COVID-19-related policies. The Oxford COVID-19 Government Response Tracker (OxCGRT) compiles policies related to economics, containment, and health for 180 countries and most US states [7]. OxCGRT focuses on public health mitigation but does not include many relevant economic and safety net policies. Additionally, the COVID-19 US State Policy database (CUSP) at Boston University captures state policies related to COVID-19 prevention, economic precarity, and health equity does not include U.S. federal policies [8].

The current study addresses this critical knowledge gap through the creation of a comprehensive dataset that systematically documents policy changes across four categories relevant to health and social equity during the pandemic. This database is publicly available, enabling researchers, policymakers, and other stakeholders to rigorously assess the short- and long-term effects of these policies and inform future preparedness response plans in California and beyond.

## 2. Materials and Methods

We generated a comprehensive database to capture the policy landscape of California during the COVID-19 pandemic from March 2020 to November 2021. First, we generated a list of California and federal policies based on the following inclusion criteria: (1) The policy/program had been modified or created in response to COVID-19 or in response to pandemic-related social or economic hardships; (2) the policy/program was enacted in the state of California or at the federal level and was implemented in California; and (3) the selected programs/policies fell into one of four domains: (1) COVID-19 mitigation; (2) safety net; (3) housing; and (4) school/childcare. These categories were chosen given their relevance to health and social equity and their integral role in achieving healthier and more equitable communities, as documented in prior literature [13].

Next, from July-November 2021, we searched California and federal government websites for programs and policies of interest and systematically documented the following information. For each policy we found: what changes occurred, when these changes occurred, whether the policy was implemented at the national or state level, relevant legislative documentation, announcement dates, coverage dates, eligibility criteria, and benefit amounts. One methodological innovation of this study leveraged an established digital archive tool, the Wayback Machine [14], to better collect and document historical data from archived versions of government websites. When websites only provided current policy data and had removed outdated information, we used the Wayback Machine to access archived versions of these websites. This tool allowed us to create a more robust longitudinal policy database, even when searching for data on programs which had changed multiple times or even expired. When government web pages did not exist or did not provide the relevant program details required for our database, we used the search engine Google to find press releases, social media posts from official sources (e.g., governmental organizations), and news sources from the time of the policy change. Search terms included names and acronyms of a program/policy and restriction of the search date range to 1 month before and 1 month after the program/policy change of interest. The search term “California” was included when searching for information on state-level policies. For documentation and replication purposes, our team archived all websites used for this database that were not already archived by The Wayback Machine, avoiding the potential loss of relevant policy information in this rapidly evolving policy environment. Current and historical citations for each policy were embedded in the database itself. To ensure quality and validity of our work, three data collectors independently reviewed the information included in the dataset and all abstracted policy details using identified citations. Discrepancies were discussed and resolved between data collectors.

After the database had been compiled, we tabulated the number of policies in each domain and plotted them on a timeline to examine temporal trends in policy enactment.

## 3. Results

**Figure 1:**
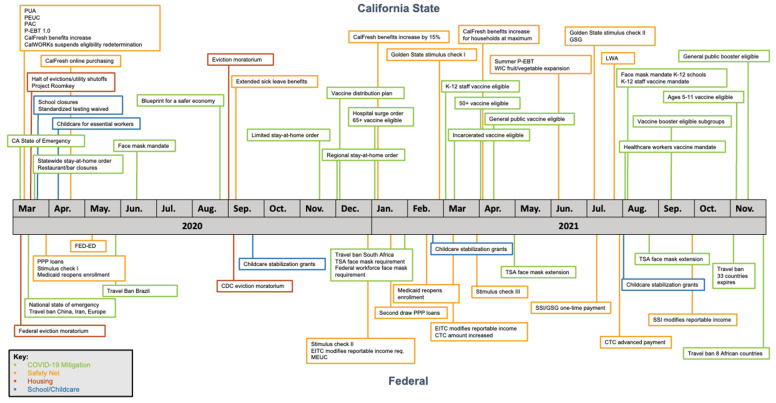

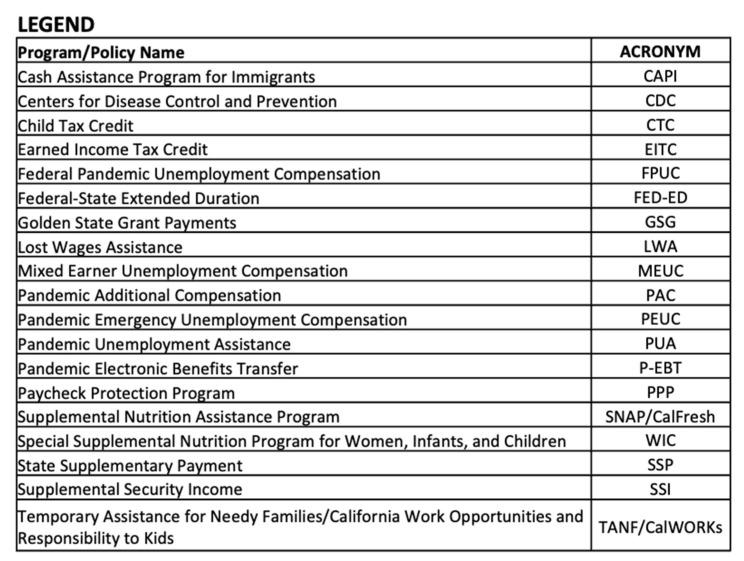
Timeline of California and federal implementation of COVID-19-related programs and policies relevant to vulnerable populations, March 2020 to November 2021.

We identified and collected data on 39 federal and California state programs and policies, including 11 COVID-19 mitigation policies, 20 safety net policies, 4 school/childcare policies, and 4 housing policies (Supplemental Table S1). Figure 1 provides a timeline of these policies, demonstrating that numerous California and federal policies were implemented concurrently with the onset of the pandemic in March 2020. As the pandemic progressed, many existing safety net programs were expanded (e.g., unemployment benefits), and new programs were formed (e.g., the Pandemic Electronic Benefits Transfer program) to continue to address evolving needs, although many have since expired.

The database also illustrates that the policy landscape was constantly changing during this time period. For example, the Supplemental Nutrition Assistance Program (SNAP, known as CalFresh in California) increased monthly allotments three times under different eligibility criteria between March 2020 and April 2021 (Supplemental Table S1). Three different stay-at-home orders were implemented in California, in addition to a tiered re-opening strategy—the “Blueprint”—guiding counties in their policymaking based on local COVID-19 burden (Supplemental Table S1). These examples, among others, highlight that just within the state of California, unique populations were differentially impacted by this rapidly changing and diverse policy landscape.

Interested investigators can also access the full database and associated documentation in the GitHub repository (https://github.com/SPHERE-UCSF/CA_COVID-19_Policy_Landscape) (accessed on 24 February 2022).

## 4. Discussion

This study synthesized both federal level and state level policy changes that were intended to alleviate the health and economic consequences of the pandemic in California. In a novel database that we made publicly available, we documented a rich policy landscape with numerous policies intended to address disease transmission and others addressing multiple social determinants of health that were impacted by the pandemic, including income, housing, food security, and employment [15,16]. Notably, we found few school/childcare policies created or expanded during the first two years of the pandemic compared with other policy categories. This apparent shortage of programs may explain why childcare has been a continuing challenge for families and in women in particular as the pandemic has progressed [17].

Our documentation of this rapidly changing policy landscape can be used in future policy evaluations to investigate the downstream health implications of pandemic-related state and federal policies. Such analyses are imperative for governments as they continue to confront additional COVID-19 surges and prepare for future public health emergencies, all while assuring health equity. This database provides the basis for such analysis in California and should be leveraged for future policy analyses or form the basis for policy data collection in other U.S. states or countries. Furthermore, documenting the dynamic and multiple changes to policies over time during the pandemic helps to illustrate the ongoing uncertainty and fluid nature of support provided during an unstable time. Future studies should assess the effects of these many changes on the intended beneficiaries of the policies.

Some policy evaluations conducted early in the COVID-19 pandemic reported mixed effects of public policies on physical and mental health. For example, local stay-at-home orders were associated with increased depression/anxiety [4], while receipt of pandemic unemployment insurance was associated with fewer symptoms of depression/anxiety and reduced healthcare delays [18]. Results of studies like these that focused on individual policies need to be interpreted in the context of the multiple co-occurring policies that we document here to account for possible confounding; that is, our database will help place these studies in the larger context of federal and state COVID-19-related policymaking in which individual policies occurred [19].

### Limitations

This database is not an exhaustive dataset of all policy changes across all sectors of government in California and nationally, but rather, a presentation of COVID-19-related policy changes at the state and federal level that are relevant to health and social equity in California during the pandemic. Nevertheless, the federal policies included in our database are still relevant for vulnerable groups in other states, and this database can be used in conjunction with OxCGRT and CUSP to create a more holistic picture of the policy landscape during this period. Additionally, due to heterogeneity in policy implementation locally, future work should also document policy changes at county or city levels. Finally, this database captures policy implementation rather than real-world enforcement (e.g., program take-up or adherence).

## 5. Conclusions

The COVID-19 pandemic has illuminated the critical nexus between public policy and public health and the implications that policy change has for public health and health inequities. We provide documentation of COVID-19-related policies in the most populous U.S. state, California, including mitigation measures related to COVID-19, as well as social policies to address the economic fallout. The granular detail we document in this database can form the basis for analyses that leverage variation to assess the health effects of COVID-19-related policies on a wide array of health outcomes [20,21,22]. This evidence will enable future policy evaluations to quantify the impact of policy changes on health disparities and inform potential interventions to address inequities.

## Data Availability

A publicly available dataset was created in this study. This data can be found here: https://github.com/SPHERE-UCSF/CA_COVID-19_Policy_Landscape (accessed on 24 February 2022).

## Supplementary Materials

The following are available online at https://github.com/SPHERE-UCSF/CA_COVID-19_Policy_Landscape (accessed on 24 February 2022).
